# Endoscopic submucosal injection of adipose-derived mesenchymal stem cells ameliorates TNBS-induced colitis in rats and prevents stenosis

**DOI:** 10.1186/s13287-018-0837-x

**Published:** 2018-04-10

**Authors:** Eduardo Martín Arranz, María Dolores Martín Arranz, Tomás Robredo, Pablo Mancheño-Corvo, Ramón Menta, Francisco Javier Alves, Jose Manuel Suárez de Parga, Pedro Mora Sanz, Olga de la Rosa, Dirk Büscher, Eleuterio Lombardo, Fernando de Miguel

**Affiliations:** 1 Gastroenterology Department, La Paz University Hospital, Paseo de la Castellana 261 4th floor, 28046 Madrid, Spain; 2 Cell Therapy Laboratory, La Paz Hospital Institute for Health Research, Madrid, Spain; 3 Tigenix SAU, Tres Cantos, Madrid, Spain; 4 Pathology Department, La Paz University Hospital, Madrid, Spain; 5 Grifols SA, Sant Cugat del Vallés, Barcelona, Spain

**Keywords:** Inflammatory bowel disease, Cell therapy, Mesenchymal stem cells, Endoscopic treatment

## Abstract

**Background:**

Mesenchymal stem cells have potential applications in inflammatory bowel disease due to their immunomodulatory properties. Our aim was to evaluate the feasibility, safety and efficacy of endoscopic administration of adipose-derived mesenchymal stem cells (ASCs) in a colitis model in rats.

**Methods:**

Colitis was induced in rats by rectal trinitrobenzenesulfonic acid (TNBS). After 24 h ASCs (10^7^ cells) or saline vehicle were endoscopically injected into the distal colon. Rats were followed for 11 days. Daily weight, endoscopic score at days 1 and 11, macroscopic appearance at necropsy, colon length and mRNA expression of Foxp3 and IL-10 in mesenteric lymph nodes (MLN) were analyzed.

**Results:**

Endoscopic injection was successful in all the animals. No significant adverse events or mortality due to the procedure occurred. Weight evolution was significantly better in the ASC group, recovering initial weight by day 11 (− 0.8% ± 10.1%, mean ± SD), whereas the vehicle group remained in weight loss (− 6.7% ± 9.2%, *p* = 0.024). The endoscopic score improved in the ASC group by 47.1% ± 5.3% vs. 21.8% ± 6.6% in the vehicle group (*p* < 0.01). Stenosis was less frequent in the ASC group (4.8% vs. 41.2%, *p* < 0.01). Colon length significantly recovered in the ASC group versus the vehicle group (222.6 ± 17.3 mm vs. 193.6 ± 17.9 mm, *p* < 0.001). The endoscopic score significantly correlated with weight change, macroscopic necropsy score and colon length. Foxp3 and IL-10 mRNA levels in MLN recovered with ASC treatment.

**Conclusions:**

ASC submucosal endoscopic injection is feasible, safe and ameliorates TNBS-induced colitis in rats, especially stenosis.

**Electronic supplementary material:**

The online version of this article (10.1186/s13287-018-0837-x) contains supplementary material, which is available to authorized users.

## Background

Inflammatory bowel disease (IBD) includes Crohn’s disease (CD) and ulcerative colitis (UC); it is characterized by chronic and relapsing inflammation of the intestinal tract. Its pathogenesis is not completely understood, but a dysregulation of the innate and adaptive immune system, genetic influences and environmental factors are suggested to intervene in the development of the disease. With current treatments, there is still a substantial proportion of patients in whom remission cannot be achieved, leaving unmet needs in the treatment of IBD and leading to the emergence of new treatments [1].

Mesenchymal stem cells (MSCs) are cells that can be isolated from several adult tissues, including bone marrow (BM-MSCs) and adipose tissue (ASCs) [2]. Adult MSCs have been proposed as a potential treatment for several diseases, including immune-based treatments, due to their multilineage differentiation capabilities that could allow MSCs to repair damaged tissues [1, 3] and their capacity to modulate the function of the majority of immune cells [2, 4]. This treatment could promote the regulation of the inflammatory cascade by inducing tolerogenic phenotypes in antigen-presenting cells (APCs) [5] while inhibiting the proliferation of cytotoxic T-cells and promoting differentiation toward regulatory phenotypes in lymphocytes (regulatory T cells) and macrophages (M2 macrophages) [6]. The immunomodulatory properties of MSCs rely not only on cell-to-cell interactions but also on effects mediated by a variety of soluble factors [7–9].

MSCs are considered to have low immunogenicity due to a low expression of major histocompatibility complex (MHC)-I and an absence of MHC-II and classic costimulatory molecules, allowing allogeneic use in the clinical setting or even xenogeneic use for research purposes [10, 11].

Regarding IBD cell therapy, MSCs from different sources have reported efficacy through various routes of administration (systemic and local) in animal studies on experimental colitis [12]. Thus, MSC treatment reduced the clinical and histopathological severity in experimental animal models of colitis by downregulating the Th1 and Th17-driven inflammatory response (i.e. reduction of pro-inflammatory cytokines such as tumor necrosis factor (TNF), interferon gamma (IFNγ), interleukin (IL)-6, IL-12 or IL-17), increasing the levels of anti-inflammatory cytokines such as IL-10, and promoting the generation of immune cells with immunomodulatory properties such as FoxP3 regulatory T cells, regulatory B cells, and M2 macrophages [13–18]. The engraftment of MSCs on the inflamed gut might play an important role for their therapeutic effect on IBD models [13, 14, 19, 20] Interestingly, preactivation of MSCs with inflammatory mediators such as IFNγ, IL-1β, IL-25 or poly (I:C) increase their therapeutic capacity in vivo by increasing their immunomodulatory properties and capacity to home to the site of inflammation [21–24]. Noteworthy, efficacy on the treatment of experimental colitis has been also obtained using components of MSCs, such as conditioned medium and extracellular vesicles, showing that MSC secreted factors can ameliorate colitis [25, 26]. Another aspect of interest is that exposure of MSCs to drugs commonly used in the treatment of IBD do not appear to affect MSC function in vitro [27, 28]. Furthermore, MSC-based therapies have been already used in human studies on fistulizing [29–31] and luminal CD [32–34]. Importantly, Panés et al., recently reported for the first time the significant efficacy of the intralesional treatment with allogeneic ASCs (Cx601) of complex perianal fistulas in Crohn’s patients, in a randomized, placebo-controlled phase III clinical trial [35].

Although promising, MSC therapy for IBD still raises some questions, such as the best route of administration to optimize bioavailability in the damaged areas. Conflicting results have been reported to date in terms of intravenous efficacy, and local approaches used in animal experiments, such as intraperitoneal inoculation or colon injection through laparotomy, are impractical for human use [36]. In this study, we propose a new administration route for IBD cell therapy: injecting cells into the colon submucosa using endoscopy, allowing direct cell delivery to the damaged area, while enabling the evaluation of disease severity and evolution.

The aim of this study was to evaluate the feasibility, safety and efficacy of submucosal endoscopic injection of human ASCs in a 2,4,6-trinitrobenzenesulfonic acid (TNBS)-induced colitis model in rats, and the utility of endoscopy to evaluate and follow the course and severity of the disease over time.

## Methods

### Ethics

All the animal experiments were performed following approval from the Animal Experimental and Welfare Ethics Committee of La Paz University Hospital (CEBA 24-2010), and in accordance with the guidelines of the directive 2010/63/EU from the European Parliament and of the Council on the protection of animals used for scientific purposes and the corresponding Spanish regulations (RD53/2013).

### Animals

Immunocompetent SD-OFA male rats (Charles River, Barcelona, Spain), weighing 375–400 g were used, and were kept in standard stabulation conditions (12 h light/dark daily cycle) in our facilities with pellet chow and water ad libitum throughout the experiment.

### ASC isolation

For ASC isolation, lipoaspirates were obtained from human adipose tissue after informed consent from otherwise healthy adult male and female donors undergoing cosmetic surgery.

The ASC isolation was performed as previously reported [37]. Briefly, the lipoaspirates were washed twice with phosphate-buffered saline (PBS) to remove contaminating debris and red blood cells and digested at 37 °C for 30 min with 0.075% collagenase (Type I, Invitrogen, Carlsbad, CA, USA) in the PBS. The digested sample was washed with 10% fetal bovine serum (FBS), treated with 160 mM ammonium chloride to eliminate the remaining red blood cells, suspended in culture medium (Dulbecco’s modified Eagle medium), containing 10% FBS, and filtered. The cells were seeded onto tissue culture flasks and expanded at 37 °C and 5% carbon dioxide. The culture medium was changed every 3 to 4 days. The cells were passed to a new culture flask when the cultures reached of confluence, and were phenotypically characterized according to their capacity to differentiate into chondro-, osteo-, and adipogenic lineages [38, 39]. In addition, ASCs were verified by flow cytometry staining for specific surface markers: positive for HLA-I, CD73, CD90 and CD105; and negative for HLA-II, CD14, CD19, CD34 and CD45. A pool of six various ASC samples from male and female donors (population doubling 12–14) were used in the study.

### Experimental design

On day 0, the rats were weighed and colitis was induced. On day 1, the animals that presented weight loss underwent endoscopic evaluation under anesthesia. After colonoscopy, treatment was subsequently applied according to the assigned experimental group: ASC group (*n* = 25), or vehicle (PBS, *n* = 21). Two additional groups were used: one without colitis induction was used as a healthy control (*n* = 25); and another with induced colitis but without endoscopy or treatment, the TNBS group (*n* = 13), was used as a safety control for the endoscopy and injection.

All the animals were weighed daily. On day 11, a second colonoscopy was performed under anesthesia for the assessment of colonic damage; blood was then obtained by cardiac puncture, and the animals were euthanized with saturated potassium chloride through intracardiac injection. A medial abdominal incision was then performed for macroscopic evaluation.

### Colitis induction

On day 0, the animals were weighed and anesthetized with inhaled isofluorane (5% induction and 2% maintenance), and feces were removed by gentle manual pressure of the abdomen. While in a supine position, a flexible plastic intravenous catheter (BD Insyte™ Autoguard™ 18G, Becton Dickinson, Madrid, Spain) was inserted 5 cm from the anal verge, and a single bolus of 0.5 ml of TNBS (Sigma-Aldrich, Tres Cantos, Spain), 30 mg/ml diluted in 50% ethanol, freshly prepared, was delivered slowly. The rats were kept in a head-down position for 1 min to prevent immediate expulsion of TNBS, and were then returned to their cages where they recovered consciousness shortly thereafter [40, 41].

### Endoscopy

Approximately 24 h after colitis induction, the animals were weighed and anesthetized with inhaled isofluorane. Prior to the endoscopy, colon cleansing was performed with a 20-ml room temperature (RT) saline solution enema.

The endoscopy was performed with a videoendoscope GIF-XP-160 (Olympus Optical Co Ltd, Tokyo, Japan), with an outer diameter of 5.9 mm, 180°/90° up/down bending, 100°/100° right/left bending, 103 cm working length, 120° view field, 2 mm working channel and a CV-145 processor (Olympus Optical Co Ltd).

While in the supine position, the endoscope was inserted into the rectum, advancing until the splenic flexure (8–10 cm). All the endoscopies were digitally recorded for posterior analysis by two different observers.

To assess colitis severity, we developed an endoscopic index, adapted from published animal endoscopic experiments [19, 42–45] and human IBD scales [46–48]. The degree of inflammation, ulceration, stenosis, thickening, bleeding and extent of disease were scored individually and a final score was obtained by adding all the variables, ranging from 0 to 25 (Table 1).

**Table 1:** Endoscopic score. The score is the result of the sum of each item

Inflammation	Ulceration type	Stenosis	Colon thickening	Spontaneous bleeding	Inflammation longitudinal extent (cm)
0 Normal aspect of the mucosa	0 No ulcers	0 No stenosis	0 Transparent	0 Absent	
1 Hyperemia	1 Cicatricial ulcer	1 Mild stenosis	1 Mild	1 Present	
2 Ulceration, covering < 1/4 of the circumference	2 Superficial ulcer	2 Stenosis preventing endoscope pass	2 Pronounced		
3 Ulceration, covering 1/4 to 1/2 of the circumference	3 Medium depth ulcer		3 Totally opaque		
4 Ulceration, covering > 1/2 of the circumference	4 Deep ulcer				
5 Circumferential ulceration	5 Necrotic ulcer				
6 Circumferential ulceration+ longitudinal extension					

### Cell injection

Endoscopic needles (23G × 5 mm, 160 cm length, Olympus Optical Co Ltd) were used for submucosal injection. Following endoscopic evaluation, a needle was passed through the endoscope’s working channel, then introduced tangentially into the submucosa using the dynamic endoscopic submucosal injection method [49], injecting 0.2 ml PBS containing ASCs or not (total dose 10^7^ ASCs) in four different spots surrounding the lesions. This dose and number of injections were chosen taking into account the maximum concentration in which ASC viability was guaranteed and covering the extension of the damaged colon while keeping a low volume of fluid, and thus avoiding potential mucosal damage caused by over injection.

The complete procedure, including submucosal injections, usually took no longer than 10 min.

### Macroscopic evaluation

After euthanasia, the abdominal cavity was exposed through medial abdominal incision, allowing visualization of the colon and the presence of adherences. Photographs were taken for evaluation by two different observers. Macroscopic damage was assessed by the degree of colon wall vascularization, wall thickening and presence of adherences, for a final score ranging from 0 to 9 (Table 2).

**Table 2:** Macroscopic damage score. The score is the result of the sum of each item

Colon wall vascularization	Colon wall thickening	Adherences to the colon wall
0 Normal	0 Normal	0 Absence
1 Mild vascular pattern distortion	1 Mild	1 Mild adherences
2 Pronounced vascular pattern distortion	2 Pronounced	2 Moderate adherences
3 Complete absence of vascular pattern	3 Very intense	3 Pronounced adherences

The colon was removed to measure its entire length from the colocecal junction to the anal verge, and the distal part excised and fixed in buffered formalin.

Mesenteric lymph nodes (MLN) were collected, snap-frozen in liquid nitrogen and stored at − 80 °C for further use.

### Immunohistochemistry

Distal colonic tissue was fixed in 10% buffered formalin, embedded in paraffin, cut into 5-μm sections and stained with hematoxylin-eosin.

To detect ASCs by immunohistochemistry, tissue sections were dewaxed and rehydrated, and then microwaved for 20 min in 0.01 M trisodium citrate buffer pH 6.0. After cooling to RT, endogenous peroxidase was blocked by incubating the tissues for 10 min in 1% H_2_O_2_ in methanol. After rinsing with TBST buffer, the samples were blocked for 1 h at RT with 5% normal goat serum and 1% bovine serum albumin in TBST, then incubated overnight at 4 °C with anti-human mitochondria antibody (113-1, Abcam, Cambridge, UK), 1:500 in blocking solution. Detection was performed with goat anti-mouse biotinylated IgG (Life Technologies, Carlsbad, CA, USA) 1:250 in blocking solution for 1 h at RT, followed by the ABC kit (HRP-based, Vector Laboratories, Burlingame, CA, USA), for 1 h at RT, and DAB as the chromogen. Sections were counterstained with hematoxylin, and mounted with DPX.

### RNA isolation, reverse transcription-polymerase chain reaction (RT-PCR), quantitative PCR

Total RNA was isolated from frozen MLN with TRIzol reagent (Ambion, Life Technologies), according to the manufacturer’s recommendations. The average yield was 1.91 ± 0.06 μg RNA per mg MLN. Complementary DNA synthesis was performed with MultiScribe Reverse Transcriptase and oligo (dT)_16_ (Applied Biosystems, Foster City, CA, USA), according to the manufacturer’s recommendations. Standard PCR was performed with 1u DNA polymerase (Biotools, Madrid, Spain) and 0.5 μM each oligonucleotide for 35 cycles of 30 s at 95 °C, 30 s at 60 °C and 30 s at 72 °C. The sequences of the oligonucleotides were as follows: Foxp3, forward, 5’-CAG CTG CCT ACA GTG CCC CTA G-3′, reverse, 5’-CGT TTG CCA GCA GTG GGT AG-3′; IL-10, forward, 5′-GGA TCC AAC GCA GCC TTG CAG AAA C-3′, reverse, 5’-ACG CGT ATT TTT CAT TTT GAG TGT CAC GTA GGC -3′; ß-Actin, forward, 5′-AGA GGG AAA TCG TGC GTG-3′, reverse, 5’-CTG GGT ACA TGG TGG TGC-3′. The PCR products were analyzed on 1.6% agarose gels in 0.5 × TBE buffer (Tris-Borate-EDTA) containing 1 × REALSafe (Durviz, Valencia, Spain) and with a digital photodocumentation system (Alliance 2.7, UVitec, Cambridge, UK).

For the quantitative PCR, we used a CFX96 Touch system (Bio-Rad, Hercules, CA, USA) and Quantimix Easy kit (Biotools, Madrid, Spain) containing 0.3 μM each oligonucleotide and 0.5 × SYBR Green (Life Technologies), for 40 cycles of 20 s at 95 °C, 20 s at 60 °C, 30 s at 72 °C and 2 s at 80 °C (when fluorescence was acquired). The oligonucleotide sequences were as follows: Foxp3, forward, 5′-TGG CAG GGA AGG AGT GTC AG-3′, reverse, 5’-GGC TGA CTT CCA AGT CTC GT-3′; IL-10, forward, 5’-GGC TCA GCA CTG CTA TGT TGC C-3′, reverse, 5’-AGC ATG TGG GTC TGG CTG ACT G-3′; GAPDH, forward, 5′- CGT GGA GTC TAC TGG TGT CTT CAC C-3′, reverse, 5′- GAT GGC ATG GAC TGT GGT CAT GAG C-3′. We used the standard 2^-ΔΔCt^ method to quantitate expression levels.

### Statistics

The statistical analyses were performed with SPSS 20.0 (SPSS Inc., Chicago, IL, USA) and Prism 5.01 (GraphPad Software Inc., San Diego, CA, USA). The statistical level of significance was *p* < 0.05, corrected for multiple comparisons where appropriate.

The quantitative data are expressed as mean ± SD.

The differences between continuous and qualitative variables were calculated with non-parametric tests: the Kruskal-Wallis or the Mann-Whitney *U* test*.* The Wilcoxon signed-rank test was used for paired analysis.

For the frequency analysis between qualitative variables we used the chi-squared test or Fisher’s exact test, when necessary (if *n* < 20 or any value in the expected value table was < 5).

Correlations were analyzed by Pearson’s correlation coefficient. Slopes of linear regression were compared with the beta coefficient of the simple linear regression and its standard error.

Survival curves were made using the Kaplan-Meier method and the log-rank test.

## Results

### Basal characteristics

Twenty-four hours after a TNBS enema, the rats suffered significant weight loss (− 3.9% ± 2.4% vs. 1.0% ± 1.4% in the control group, *p* < 0.001); diarrhea and other signs of discomfort were also evident in the colitic animals. The endoscopic score increased significantly in these animals compared with the control group (16.1 ± 4.9 vs. 1.3 ± 1.9, *p* < 0.001). The selection of rats to either treatment group, PBS or ASC, was not biased, given neither weight loss (− 3.6% ± 2.8% vs. -3.0% ± 1.9%, respectively, *p* = 0.21) nor endoscopic score (17.6 ± 4.3 vs. 15.0 ± 5.0, respectively, *p* = 0.08) was statistically different.

### Feasibility and safety

**Fig. 1:**
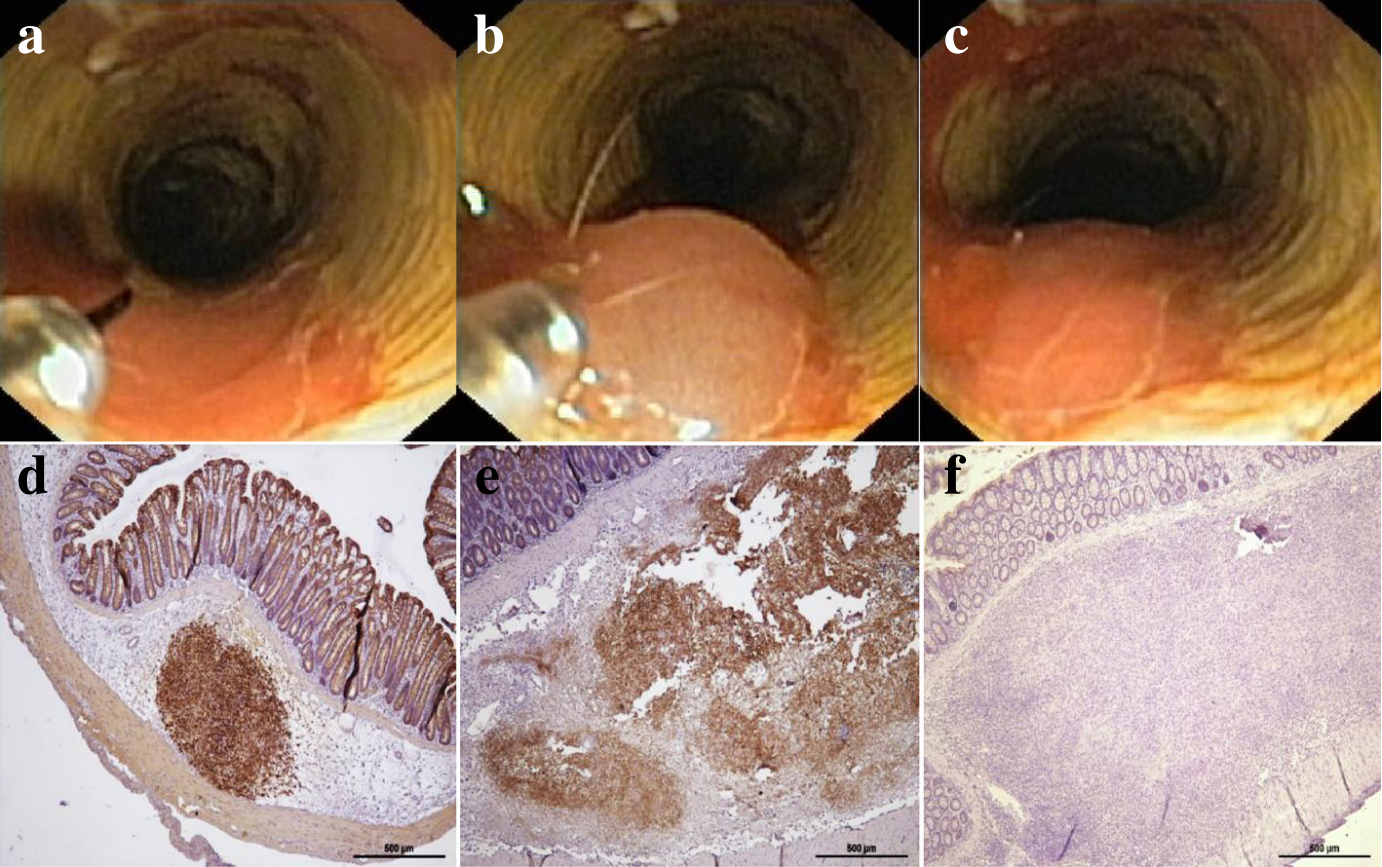
Injection feasibility and cell location. Cells were injected into the submucosa using a 23G endoscopic needle. **a**, **b** and **c** injection procedure, bleb formation and endoscopic appearance after injection. Cells are injected into the submucosa, this is demonstrated by presence of hASC staining (*brown*) using anti-human mitochondria antibodies in colon submucosa 24 h after injection in a healthy animal (**d**) or in a colitic animal (**e**). Eleven days after injection in a colitic animal that recovered weight cells no staining was seen (**f**)

The endoscopic injection was successful in all the rats, with formation of a visible submucosal bleb (Fig. 1a-c). Neither significant adverse events nor mortality due to the procedure occurred.

We assessed the correct location of the cell injection using immunohistochemistry with anti-human mitochondria antibody, which allows detection of human ASCs. In a healthy animal in which ASCs were injected following the same method of the experiment, a dense accumulation of positive cells was observed in the colon submucosa 24 h after the injection, with no signs of immune reaction (Fig. 1d).

In the colitic rats, we also detected ASCs in the colon submucosa 24 h after cell injection, with intense inflammatory infiltrate and edema (Fig. 1e). At the end of the experiment, however, we could not detect ASCs by immunohistochemistry within the submucosa, even in the animals that showed signs of improvement: the extensive areas that were seen in the submucosa of these animals showed cells with a fibroblastic phenotype, less inflammatory infiltrate and almost absent edema (Fig. 1f).

There was no difference in overall survival rate with either treatment, (85.7% PBS vs. 83.3% ASCs vs. 84.6% TNBS, *p* is not significant). As expected, the healthy control group suffered no deaths (data not shown).

### Weight evolution

**Fig. 2:**
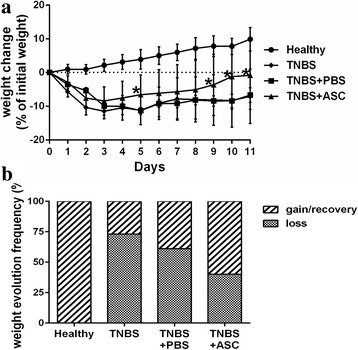
hASC improves weight evolution. Colitis induces weight loss. **a** Body weight was measured daily. Faster improvement was seen in ASC group, being significantly different on days 5, 9, 10 and 11. *p < 0.05. **b** Frequency of weight recovery by treatment group. 60% of animals treated with ASC recovered weight vs. 38.9% in PBS group and 27.3% in TNBS group (*p* = n.s.). *ASCs* adipose-derived mesenchymal stem cells, *PBS* phosphate-buffered saline, *TNBS* trinitrobenzenesulfonic acid

All the control rats gained weight steadily throughout the course of the experiment, reaching a significant increase of 9.9% ± 3.4% (*p* < 0.001) compared with their initial weight (Fig. 2a, b). Regarding the colitic rats, the TNBS group suffered rapid weight loss, reaching − 11.9% ± 2.1% at day 3; they then began a slight recovery, with mean weight loss at the end of the experiment of − 8.7% ± 8.2% (*p* = 0.021) (Fig. 2a). The PBS-treated rats also exhibited a similar pattern, with significant weight loss that reached a maximum on the fifth day after induction with the hapten (− 11.3% ± 4.2%). After this day, the rats gained weight slightly, and, on average, did not reach initial values (− 6.7% ± 9.2% on day 11, *p* = 0.024) (Fig. 2a).

The ASC-treated rats also suffered an initial weight loss that reached its peak on day 3 (− 8.4% ± 4.2%), after which, unlike the other colitic groups, weight recovery was evident, reaching, on average, initial values by the end of the experiment (− 0.8% ± 10.1% on day 11, *p* = 0.741) (Fig. 2a). In fact, the ASC-treated animals gained weight with a slope comparable to the one observed in the control group (0.93 ± 0.08 vs. 0.89 ± 0.04, respectively, *p* = 0.601), whereas the slopes for the TNBS and the PBS-treated groups were significantly flatter (0.52 ± 0.09, *p* = 0.001, and 0.41 ± 0.09, *p* < 0.001, respectively) (Fig. 2a).

Weight recovery was statistically significant in the ASCs group over the PBS group on day 5 (− 6.5% ± 5.86% vs. −11.32% ± 4.18%, respectively, *p* = 0.009), day 9 (− 3.7% ± 9.1% vs. −8.5% ± 6.8%, respectively, *p* = 0.034), day 10 (− 1.9% ± 9.4% vs. −8.4% ± 8.6%, respectively, *p* = 0.013) and day 11 (−0.8% ± 10.1% vs. −6.7% ± 9.2%, respectively, *p* = 0.037).

Individually, some animals from every colitic group recovered initial weight by the end of the experiment, but the frequency of weight recovery was higher in the ASC-treated animals compared with the TNBS and PBS groups: 60.0% (12/20), 27.3% (3/11) and 38.9% (7/18), respectively (*p* is not significant) (Fig. 2b).

### Endoscopic analysis

**Fig. 3:**
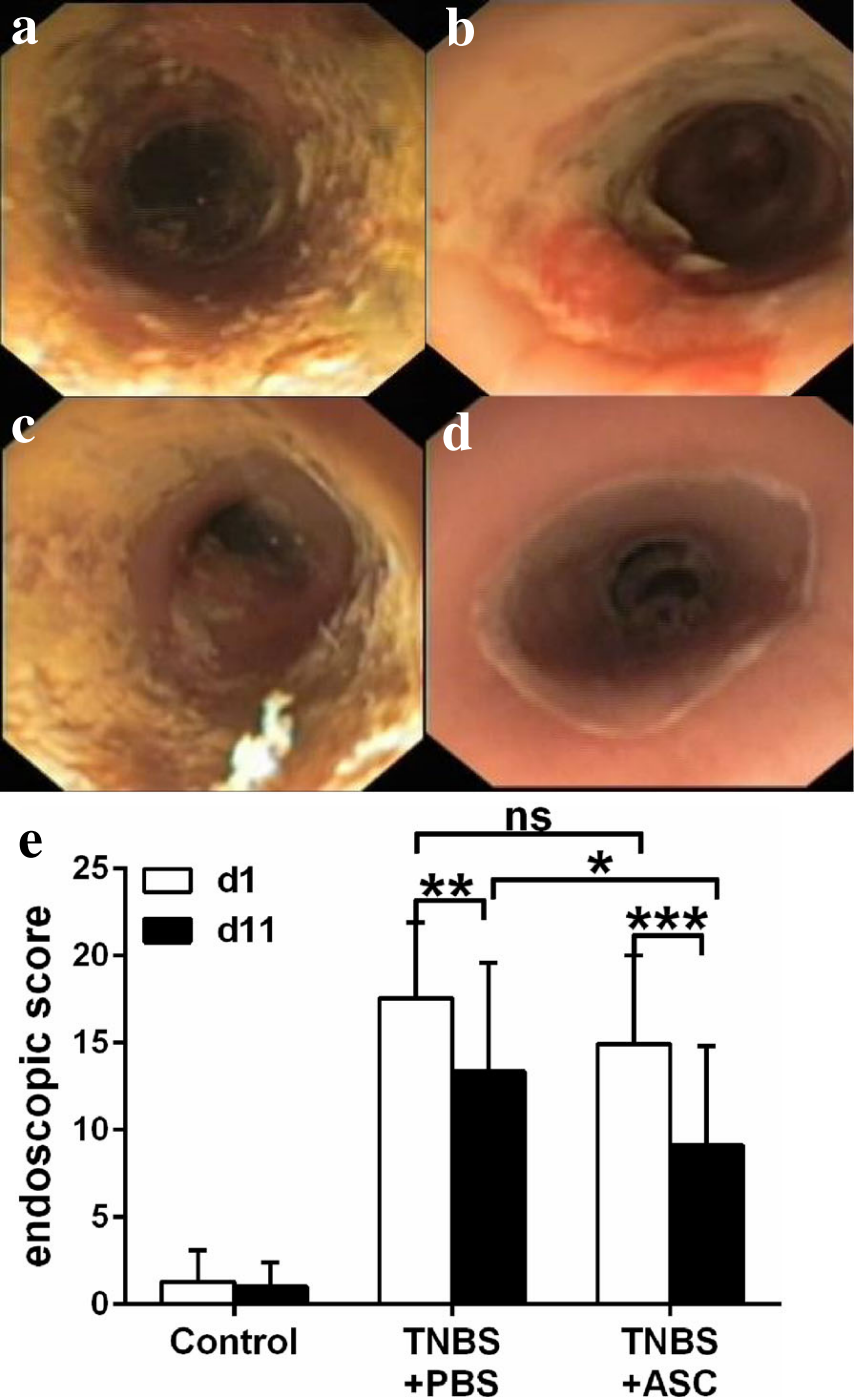
Endoscopic evaluation of the colitis. TNBS induced distal colitis characterized by erythema, edema, loss of vascular pattern and ulceration. This is still evident after 11 days of the onset of the disease. **a**-**b** Endoscopic evolution of a PBS-treated rat at day 1 and 11. **c**-**d** Endoscopic evolution of an ASC-treated rat at day 1 and 11. **e** Evolution of the endoscopic score over time per treatment group. The endoscopic score at baseline was not different between groups. As expected this score improved between day 1 and 11 in both groups. The change in ASC group was greater than in the PBS group (−6.6 ± 2.9 vs. −3.5 ± 5 points, *p* = 0.011). Score at day 11 did not achieve significant difference between groups *p* = 0.052. **p < 0.01, ***p < 0.001. *ASCs* adipose-derived mesenchymal stem cells, *PBS* phosphate-buffered saline, *TNBS* trinitrobenzenesulfonic acid

After colitis induction, endoscopic signs of damage were evident, with disappearance of vascular pattern, edema and ulceration (Fig. 3a, b), which improved over time, in particular in the ASC group (Fig. 3d).

We evaluated the endoscopic mucosal damage following the score described in Table 1, with higher scores for more severe inflammation.

There is no validated score for endoscopic evaluation of experimental colitis severity. The most commonly used variable for follow-up is daily weight; therefore, we first analyzed the overall correlation between our colonoscopy score and weight change, finding a strong correlation at both time points evaluated: at day 1, the Pearson coefficient was *r* = − 0.75 (*p* < 0.001) and at the end of the experiment at day 11 *r* = −0.78 (*p* < 0.001) (Additional file 1: Figure S1).

At day 11, the final endoscopic score was 9.1 ± 5.6 in the ASC group compared with 13.3 ± 6.2 in the PBS group (*p* = 0.052) (Fig. 3e). As expected in an acute colitis model, both groups improved; however, this improvement was significantly greater in the ASC group compared with the PBS group (−6.6 ± 2.9 vs. −3.5 ± 5 points, respectively, *p* = 0.011). This represents a mean improvement of 47.1% ± 5.3% in the ASCs compared with 21.8% ± 6.6% in the PBS group (*p* = 0.005).

**Fig. 4:**
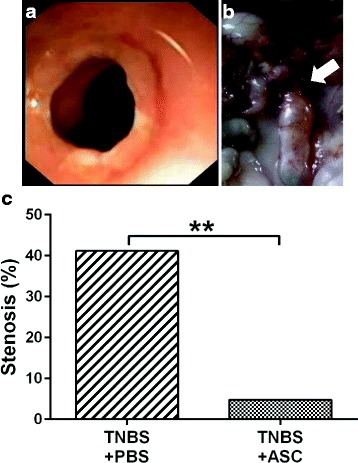
Stenosis development is decreased with ASC treatment. **a** Endoscopic image from a PBS-treated rat with stenosis. **b** Macroscopic aspect of the colon of the same rat. The stenosis is marked with a *white arrow*. **c** Frequency of stenosis in the PBS and ASC groups. **p = 0.01. *ASCs* adipose-derived mesenchymal stem cells, *PBS* phosphate-buffered saline, *TNBS* trinitrobenzenesulfonic acid

ASC treatment dramatically decreased the development of stenosis (defined as the presence of a narrowing of the lumen that hinders or prevents passage of the endoscope) when evaluated by endoscopy on day 11 (Fig. 4a, b). While in the PBS group, 7 of 17 evaluable rats (41.2%) developed stenosis, whereas only 1 of 21 rats (4.8%) did so in the ASC group (*p* = 0.001) (Fig. 4c).

### Macroscopic evaluation

**Fig. 5:**
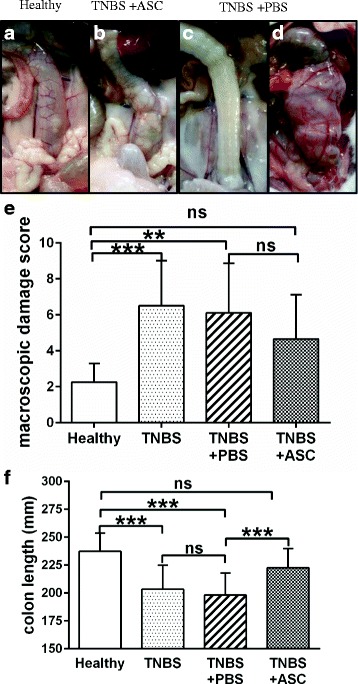
Macroscopic evaluation. Colitis is characterized by edema, loss of vascular pattern and presence of adherences. **a**-**d** Macroscopic aspect of the colon in a control rat (**a**), ASC-treated rat (**b**) and PBS-treated rats with diverse severity grades (**c** and **d**). **e** Macroscopic damage score after sacrifice per group. Higher scores represent more severe damage. The score did not achieve statistical difference between the ASC and PBS group. **p < 0.01 ***p < 0.001. f Colon length in mm per group. Colon length decreases with inflammation, the TNBS and PBS groups had significant shorter colons than the healthy colon group and the ASC-treated group. The ASC group shows recovery and is not different from the healthy control group. ***p < 0.001. *ASCs* adipose-derived mesenchymal stem cells, *PBS* phosphate-buffered saline, *TNBS* trinitrobenzenesulfonic acid

Macroscopic evaluation of the abdominal cavity was performed after euthanasia at day 11. Vascular pattern distortion, colon wall thickening and fat tissue or visceral adhesions to the colon were assessed (Fig. 5a-d). Both the TNBS and PBS groups showed a significantly higher macroscopic damage score compared with the control group (6.5 ± 2.5, *p* < 0.001; 6.1 ± 2.7, *p* < 0.01; 2.2 ± 1.0, respectively). This total score and all the evaluated subitems were lower in the ASC group than in the PBS and TNBS groups, although there was no statistical significance among them. Nevertheless, the macroscopic damage score in the ASC group (4.7 ± 2.5) decreased to values not statistically different from the control group (Fig. 5e).

**Fig. 6:**
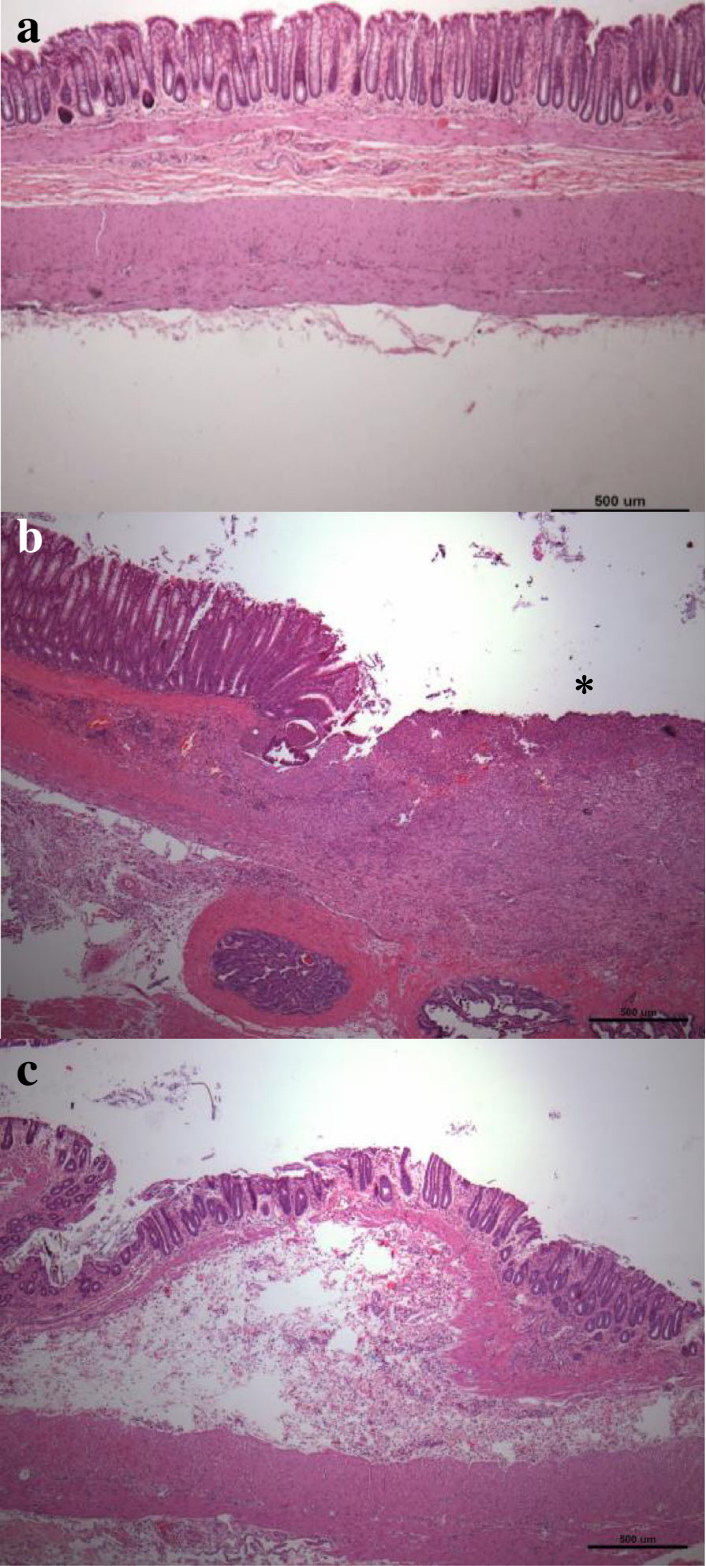
Histologic evaluation. **a** Hematoxylin-eosin staining of a control animal. **b**-**c** Sections made at the injection point of TNBS colitis at 11 days (**b**). Animal treated with PBS injection, showing mucosal ulceration * (**c**) TNBS colitis and ASC injection, showing, slight crypt distortion persists, but no ulcers or active inflammation. Scale bar 500 µm

The macroscopic damage score correlated significantly with weight change on the day of euthanasia, with a Pearson correlation *r* = 0.78 (*p* < 0.001), and with the endoscopic index on the same day (Pearson correlation *r* = 0.80, *p* < 0.001) (Additional file 2: Figure S2).

Colitis is known to induce a marked shortening of the colon. In the TNBS group and the PBS group, colon length was shorter than in the control group (204.5 ± 24.6 mm, *p* < 0.001; 193.6 ± 17.9 mm, *p* < 0.001, vs. 237.2 ± 16.3 mm, respectively). This shortening was significantly improved in the ASC group, with a mean length of 222.6 ± 17.3 mm, showing no difference from the control group (*p* = 0.2) and statistically longer than the PBS group (*p* < 0.001) (Fig. 5f). Colon length also correlated significantly with the endoscopic score at the end of the experiment, with a Pearson correlation *r* = − 0.33, *p* = 0.041 (Additional file 3: Figure S3). Distal colonic samples stained with hematoxylin-eosin were evaluated for ulceration and histologic recovery (Fig. 6).

### Immunomodulatory effects mediated by ASCs

The possible immunomodulatory effect of the local injection of ASCs into the colon submucosa was evaluated at day 11 in mesenteric lymph nodes by measuring the mRNA expression of Foxp3, a transcription factor expressed by regulatory T lymphocytes, and of IL-10, an anti-inflammatory cytokine produced by various cell types.

**Fig. 7:**
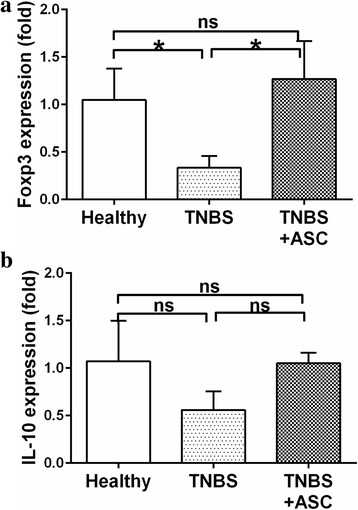
mRNA expression in MLN.ASC treatment has immunomodulatory effects. Mesenteric lymphatic nodes were isolated and frozen in liquid nitrogen. RNA was isolated and quantitative PCR was made for FoxP3 expression and IL-10. Standard 2-ΔΔCt method to quantitate expression levels was used *n* = 4. **a** Foxp3 is significantly increased in ASC treated rats (*n* = 4). **b **IL-10 expression did not reach significant difference. *p < 0.05. *ASCs* adipose-derived mesenchymal stem cells, IL-10, interleukin-10, *TNBS* trinitrobenzenesulfonic acid

As shown in Fig. 7a, Foxp3 expression decreased dramatically in the untreated colitic rats, being three times smaller in the TNBS group than in the control group (0.33 ± 0.1 vs. 1.05 ± 0.3, respectively, *p* = 0.014). The ASC group showed a recovered expression (1.27 ± 0.5 times), which was not statistically different from the control group (*p* = 0.46) (Fig. 7a).

Regarding IL-10 mRNA, the expression of IL-10 was reduced in the TNBS colitic group (0.56 ± 0.2 times) compared with the healthy control group (1.07 ± 0.4, *p* = 0.05), whereas in the ASC-treated animals, IL-10 expression was similar to the control group (1.05 ± 0.1, *p* = 0.71) (Fig. 7b).

## Discussion

In the present study, we hypothesized that local administration by endoscopy of ASCs in the submucosa of colitic rats could be an optimal route of administration to increase the bioavailability of ASCs in the damaged areas of the colon and reduce the severity of the disease.

Therefore, we evaluated the feasibility, safety and efficacy of endoscopic ASC administration in an experimental colitis model, as well as the utility of endoscopy to follow the course of the disease in rats.

Our results support endoscopy as a useful administration route for ASC treatment for colitis and its utility for assessing treatment efficacy and severity of the disease in an animal model. We also demonstrate that submucosal injection of human ASCs ameliorates the course of TNBS colitis in immunocompetent rats.

IBD pathogenesis is complex and involves multiple mechanisms. One of the most significant is the disbalance between pathogen recognition and tolerance against commensals. In this setting, MSC treatment could help restore this balance due to its diverse immunomodulatory properties, promoting both differentiation of lymphocytes and mononuclear cells toward tolerant phenotypes and suppression of activated lymphocytes [6]. Several studies have tried stem cell therapy in colitic animals with both BM-MSCs and ASCs [13–15, 19, 36, 50–54].

Although promising, MSC therapy has yet to address several questions, such as optimal doses and administration routes, selection of patients and elucidation of mechanisms of action, making animal models essential.

Regarding administration route, it has been demonstrated that ASCs have homing capabilities toward damaged areas [51, 55]. While convenient, intravenous use remains controversial due to concerns about lung entrapment that could lead to a reduced number of available cells and therefore reduced efficacy and potential adverse events [19, 52, 54, 56]. Delivering the cells directly into the damaged area would overcome this issue, and intraperitoneal use has been used in animals, but this approach is impractical in humans. Injecting cells through endoscopy could offer advantages in experimental studies and translational potential for future human use.

The TNBS chemical-induced model of colitis is inexpensive, reproducible, easy to handle and widely used [40, 41, 57]. Although human IBD is better represented by knock-out mouse models, we chose the TNBS model because it leads to distal colitis and can be used in rat strains that can grow enough to make endoscopy a feasible tool for evaluation and treatment. Therefore, even if the use of a chemical model is a limitation of our study, it is useful for the evaluation of the endoscopic administration route and that efficacy data obtained with this model is meaningful.

Our data demonstrate that endoscopy in trained hands is a simple and very effective tool for follow-up and evaluation of colitic rats, with an excellent safety profile, given we did not observe any case of perforation or significant bleeding due to the injection, and no deaths were attributed to the procedure. Moreover, there were no significant differences in the evolution of the rats with colitis without endoscopy (TNBS group) and the group with endoscopy and PBS injection. This safety profile is one of the main advantages of the technique, allowing in vivo repeated assessment of the severity and evolution of the disease, and also reducing the number of animals needed.

While it has been used for carcinogenesis [43, 58–63] and colitis [19, 42, 44, 64] evaluation, these studies are heterogeneous in aims, endoscopes and scoring systems. Although lacking a formal validation, the endoscopic score developed for this study showed a significant correlation with body weight changes, macroscopic damage and colon length, which are commonly used variables in experimental colitis. Further studies will determine which is the best score for colitis evaluation in murine models.

ASC administration demonstrated improvement in weight recovery, in colon length and in the endoscopic damage score. In addition, the macroscopic damage observed after euthanasia was numerically smaller in the treated group, although it did not reach statistical significance. These are among the main variables commonly used in colitic animals, thus supporting the ameliorating effect of the cells, and confirming the results of previous studies [13–15, 19, 36]. Although these studies differ in protocol, severity of the colitis, animal strains, cell type, dose and administration routes, all suggest a beneficial effect of ASC treatment in murine chemical-induced colitis models.

One of the main results of our study was the decrease in stenosis development; even though the experiment was not designed for this purpose, the difference between the ASC and the vehicle group is remarkable. Whereas in the PBS group stenosis developed in 41% of the animals, in the ASC group this occurred in 4.8%. This is an observation that, in our opinion, deserves further studies to elucidate whether ASCs are able to prevent stenosis development in other models and if they can also help to reverse established stenosis.

A limitation of our study is the lack of histological scoring; in our hands, microscopic changes in inflammation and healing were inconsistent.

We were able to detect the ASCs in colon submucosa using anti-human mitochondrial antibodies 24 h after injection, proving the correct location of the cells after the endoscopic administration. In agreement with previous studies reporting a short persistence of the cells in vivo, ASCs were not detected after 11 days. The fate of the ASCs in vivo remains largely unknown to date [65].

In order to determine whether the endoscopic administration of ASCs resulted in immunomodulatory effects in the colitic rats, we determined the levels of the anti-inflammatory cytokine IL-10 and the transcription factor Foxp3, characteristic of regulatory T cells. Our results show that IL-10 and Foxp3 levels in MLN of ASC-treated rats were elevated, supporting the notion that ASCs administered endoscopically have an immunomodulatory mechanism of action, similar to what has been previously described for MSCs using other routes of administration [13–18]. Nevertheless, we did not investigate in detail the mechanisms underlying the efficacy of ASCs and, therefore, further studies are needed to better understand the mechanism of action of ASCs administered through the endoscopic route of administration.

We cannot completely rule out a potential immunogenicity using xenogeneic cells; however, it appears that human ASCs are sufficiently well tolerated in this proof-of-concept model. This immunoprivileged status is probably due to a lack of MHC molecules, as has been shown in other recent studies [13–15].

If our results are confirmed there would be translational potential, in humans, endoscopic injection could be a simple, well-tolerated route of delivering cells directly into the damaged area, through a technique routinely used in patients with IBD for both diagnostic and therapeutic purposes [66], including pharmacological local injection treatment [67–69]. Cell-based therapies are currently being tested in different phases in humans, reporting efficacy of intravenous infusion with various doses and regimens of BM-MSCs, both autologous and allogeneic [32–34]. Other studies have focused on local treatment for fistulizing disease, showing improved healing with BM-MSCs [70] or ASCs [29–31, 35, 70].

## Conclusions

In conclusion, our study provides evidence that endoscopy is a safe and reliable method to administer cell therapy into the colon and to follow up colitis murine models. ASC treatment ameliorates the course of TNBS colitis and prevents stenosis development.

## Additional files

Additional file 1: **Figure S1.** Endoscopic score correlation with weight change at day 1 (A) and 11 (B). (PPTX 2289 kb)

Additional file 2: **Figure S2.** The macroscopic damage score correlates with weight change (A) and with endoscopic score (B). (PPTX 2254 kb)

Additional file 3: **Figure S3.** The endoscopic score correlation with the colon length 2. (PPTX 37 kb)

## Abbreviations

APCs: Antigen-presenting cells
ASCs: Adipose-derived mesenchymal stem cells
BM-MSCs: Bone marrow mesenchymal stem cells
CD: Crohn’s disease
FBS: Fetal bovine serum
IBD: Inflammatory bowel disease
IL: Interleukin
INF: Interferon
MHC: Major histocompatibility complex
MLN: Mesenteric lymph nodes
MSCs: Mesenchymal stem cells
PBS: Phosphate-buffered saline
RT: Room temperature
TBST: Tris-buffered saline with Tween 20
TNBS: Trinitrobenzenesulfonic acid
UC: Ulcerative colitis
