# Supplementary figures and images for: Endoscopic submucosal injection of adipose-derived mesenchymal stem cells ameliorates TNBS-induced colitis in rats and prevents stenosis
Source: Stem Cell Res Ther. 2018 Apr 10;9:95. doi: 10.1186/s13287-018-0837-x (PMC5892014; doi:10.1186/s13287-018-0837-x)

## Slide 1
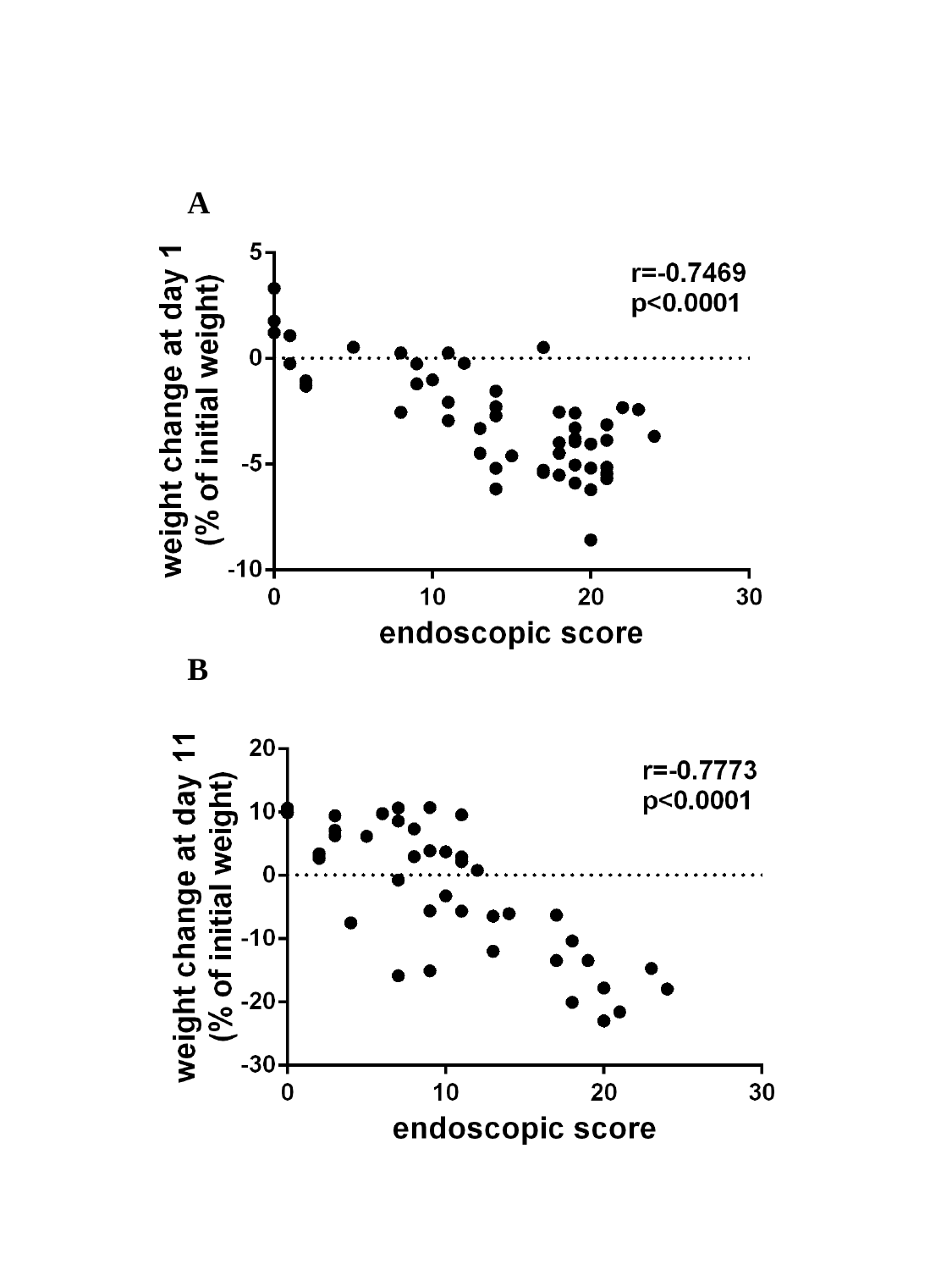

A
B

Supplement: Supplementary file 1 — Figure S1. Endoscopic score correlation with weight change at day 1 (A) and 11 (B). (PPTX 2289 kb) [file 13287_2018_837_MOESM1_ESM.pptx]

## Slide 1
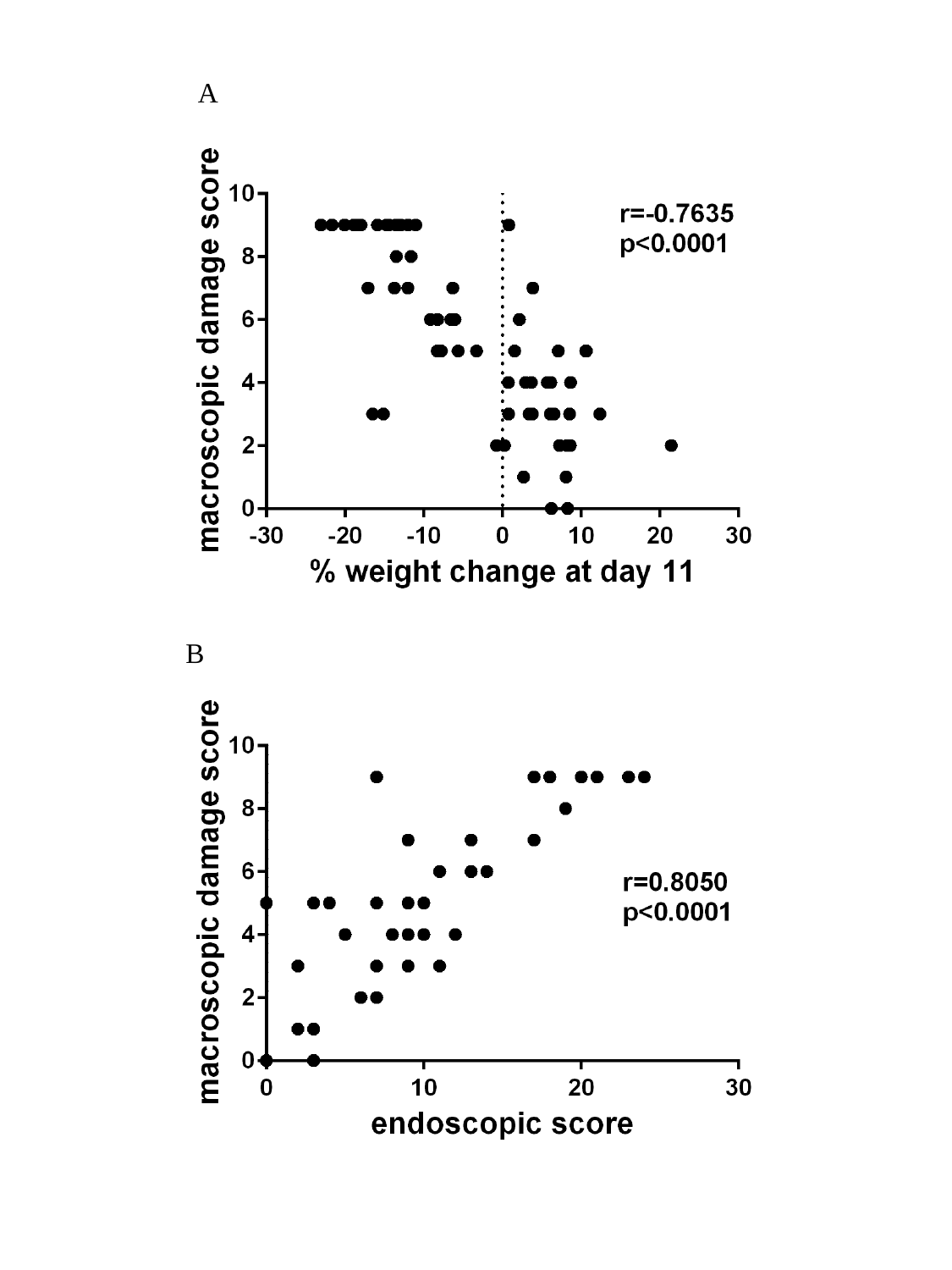

A
B

Supplement: Supplementary file 2 — Figure S2. The macroscopic damage score correlates with weight change (A) and with endoscopic score (B). (PPTX 2254 kb) [file 13287_2018_837_MOESM2_ESM.pptx]

## Slide 1
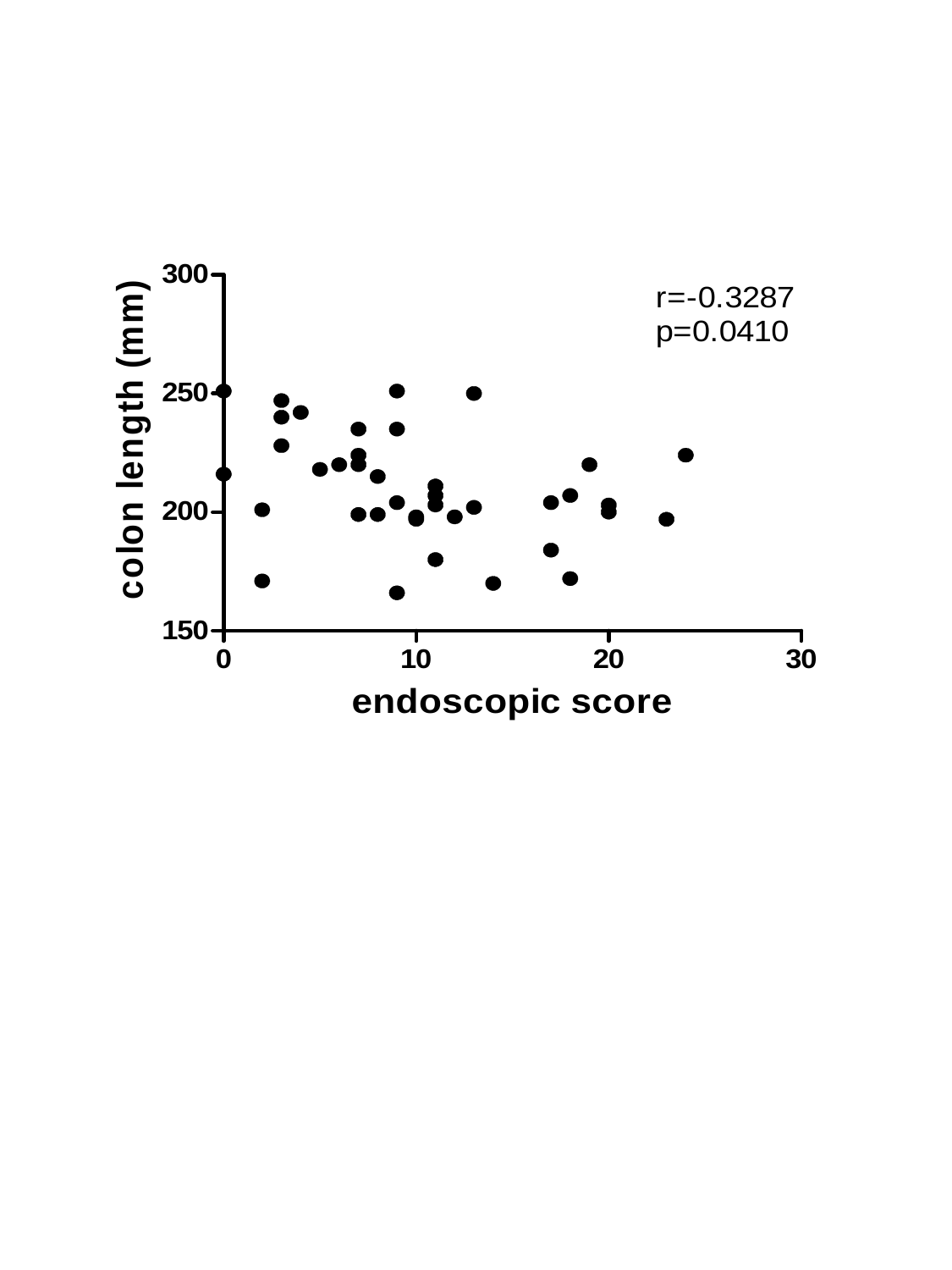

Supplement: Supplementary file 3 — Figure S3. The endoscopic score correlation with the colon length 2. (PPTX 37 kb) [file 13287_2018_837_MOESM3_ESM.pptx]
